# Advancing equity through strengthening research on health and well-being of Asian American, Native Hawaiian, and Pacific Islander

**DOI:** 10.1186/s13578-022-00834-2

**Published:** 2022-07-05

**Authors:** Dan Xi, Ming Lei, Paul Liu, Ramesh Vemuri, Yihong Ye

**Affiliations:** 1 U.S. Department of Health and Human Services, National Cancer Institute, National Institutes of Health, Bethesda, MD USA; 2 U.S. Department of Health and Human Services, National Institute of General Medical Sciences, National Institutes of Health, Bethesda, MD USA; 3 U.S. Department of Health and Human Services, National Human Genome Research Institute, National Institutes of Health, Bethesda, MD USA; 4 U.S. Department of Health and Human Services, National Institute of Aging, National Institutes of Health, Bethesda, MD USA; 5 U.S. Department of Health and Human Services, National Institute of Diabetes and Digestive and Kidney Diseases, National Institutes of Health, Bethesda, MD USA

**Keywords:** Equity, Health disparity, Minority health, Asian American, Native Hawaiian, Pacific Islander

## Abstract

The National Institutes of Health’s Asian American, Native Hawaiian and Pacific Islander Health Scientific Interest Group (NIH AANHPI-HSIG) provides a viewpoint on developing approaches to enhance research on health and wellbeing for Asian American, Native Hawaiian, and Pacific Islander ethnic populations, in order to advance racial equity amongst such populations.

## Background

Asian American (AA), Native Hawaiian and Pacific Islander (NHPI) populations are highly heterogeneous racial/ethnic groups with diverse health disparity concerns including hepatitis B and chronic liver disease, diabetes without obesity, various types of cancer, and stroke etc. [1, 2]. AA and NHPI populations also often face obstacles in seeking quality medical care. Furthermore, AA and NHPI populations are often not included in medical and public health research. Thus, there is a pressing need to develop policies and approaches to advance health equity and improve the health and well-being for AA and NHPI populations.

The National Institutes of Health Asian American Pacific Islander Health Scientific Interest Group (NIH AAPI-HSIG) was established in April 2021 with approval from Dr. Michael M. Gottesman, the NIH Deputy Director for Intramural Research [3]. The group adopted its current name AANHPI-HSIG in January 2022, after the President of United States designated “*Asian American, Native Hawaiian, Pacific Islander (AA and NHPI)”* to describe the populations in an Executive Order (14031) on May 28, 2021, which reinstated and reinvigorated the White House Initiative (WHIAANHPI) on improving the quality of life and opportunities to participate in federal programs for AAPI proclaimed in an earlier Executive Order (13125) on June 7, 1999 [4, 5]. The main goal of the group is to improve the AA and NHPI health status through advancing AA and NHPI related health research and research collaborations, mentoring junior scientists, and providing recommendations to the NIH leadership. The group has asserted leadership in providing AA and NHPI health information for research and education and a scientific exchange and collaboration platform for scientific exchange and collaboration for scientists, policy makers, and advocates to advance NIH’s mission and health equity [3].

In just over 1 year, the NIH AANHPI-HSIG has initiated several projects to integrate NIH-wide efforts to enhance knowledge and awareness of health disparity and assess the research needs for AA and NHPI populations [3, 6]. We have established NIH’s first publicly accessible scientific seminar series focusing on AA and NHPI health research and education. Since the inaugural seminar on September 23, 2021, with more than 500 registrants, several scientists presented their research and perspective on future research needs. Topic areas discussed include the AA and NHPI model minority myth and its health invisibility in scientific research, the effect of multilevel social determinants on health and the needs of multilevel approaches in research, how to strengthen clinical research and improve the quality of care including the needs of data disaggregation and the use of interpreters in language-discordant encounters for AA and NHPI populations, trusted community engagement approaches to enhance the recruitment and increase statistical power in clinical research for AA and NHPI populations, and the prevalence of lung cancer amongst the never-smoking AA and NHPI female populations and the importance of investigating the associated risk factors.

Mental health issue facing the AA and NHPI community is another important topic that was discussed at the 1st NIH AANHPI Mental Health and Well-Being Seminar Series hosted by the NIH AANHPI-HSIG [3]. As part of the effort to provide leadership, mentoring and training opportunities for the future workforce, the Mental Health seminars have been led mainly by NIH fellows since January 2022. The unique risk factors for mental health in the AA and NHPI communities were highlighted, such as low levels of psychological help seeking, gender discrimination, stigma, and interpersonal shame. The seminar on mental health and substance abuse issue for NHPI not only showcased the resilience of the NH community bolstered by the strong social support, but also called for the needs of data disaggregation, culturally proper research methods or protocols, and community-based engagement research.

Through these initiatives we have recognized that in order to advance health equity, more research on health disparities specific for the AA and NHPI racial/ethnic subgroup is needed. To address this need, a NIH-wide AANHPI Research Priority Recommendation Working Group has been formed. The Working Group led the effort to publish the first NIH Request for Information (RFI) (NOT-CA-22-047) specifically focusing on actionable priority recommendations to improve research on health and well-being of AA and NHPI populations in line with the White House [7]. Many stake holders, including professional societies, foundations, academic institutions, individuals, and companies have responded to the RFI.

To celebrate the AANHPI-Heritage Month, AANHPI-HSIG successfully organized its inaugural AA and NHPI Health Research Conference on May 4–5th, 2022, with a central theme of “From Mechanism to Translational Research: Improving the Health and Therapeutic Outcome for AA and NHPI Populations” [8]. Dr. Lawrence Tabak, the NIH Acting Director, Dr. Eliseo Perez-Stable, the Director of National Institute of Minority Health and Health Disparity, Ms. Krystal Ka‘ai, the Executive Director of the White House Initiative on Asian Americans, Native Hawaiians, and Pacific Islanders, Ms. Erika L. Moritsugu, the Deputy Assistant to the President AA and NHPI Senior Liaison, Dr. Marie Bernard, the NIH Chief Officer of Scientific Workforce Diversity, and Dr. Katrina Goddard, the Director of the Division of Cancer Control and Population Sciences representing the National Cancer Institute opened the conference with their warm remarks affirming their support for AA and NHPI health research and AANHPI-HSIG’s effort. Dr. Victor Dzau, the President of the National Academy of Medicine, and Dr. Howard Koh, a Professor of Harvard University and former Assistant Secretary of the Department of Health and Human Services presented keynote lectures.

The two-day conference, which included participants from across the HHS, had for scientific sessions on Cancer; Diabetes and Other Diseases; Other Health Conditions; Mental Health, Community Health Care, Caregiver and Indigenous Medicine, respectively; each followed by a Panel Discussion and Summary session [8]. Many issues, including the lack of research in all areas on AA and NHPI health from epidemiology, prevention, therapeutics to clinical trials and health disparities for many disease conditions (such as cancer, diabetes, heart disease, obesity, and mental health etc.) were discussed. The investigation of risk factors, etiology, molecular mutation characteristics, and mechanism of acquired resistance to therapeutics, the development of human relevant PDX model and new therapeutics, the enhancement of inclusion of more patients with driver mutations in immunotherapy clinical trial, and the engagement of international collaborative efforts for never-smoker lung cancer were considered as research needs. The effects of biological (e.g., genetic, epigenetics or gut microbiome), lifestyle (e.g., diet, exercise and sleep), environmental, behavior and other social determinants or cultural factors, and their interactions on health and response to the therapeutic outcome, besides clinical trial design including issues of data disaggregation and pharmacogenomics were addressed extensively. The importance of developing a trusted, culturally appropriate, and community-engaged collaborative research infrastructure and approaches to enhance clinical research on AA and NHPI specific health issues was recognized by all participants. Successful examples of ongoing precision medicine projects to improve ethnic inclusivity such as the NIH funded *All of Us* research project using intentional community engagement efforts and the multilevel AANHPI engagement strategy, and the New York Genome Center’s Polyethnic-1000 program that enrolls ethnically diverse and underserved cancer populations in New York City were highlighted. Participants of the conference also stressed the need of data disaggregation for AA and NHPI subgroups, discussed the language accessibility problem for AA and NHPI populations in seeking medical care or involving in clinical research, and addressed the effect of anti-Asian hate/discrimination on health. The conference not only enhanced the awareness of AA and NHPI heritage and highlighted scientific achievements in AA and NHPI health-related research, but also informed NIH leadership of the current research needs and priorities.

## Conclusions

In summary, advancing AA and NHPI health research is an integral part of the NIH‘s charge on minority health and health disparities research. The NIH AANHPI-HSIG can contribute to the WHIAANHPI on advancing equity, justice, and opportunity for AA and NHPI communities in the United States in many areas, including data disaggregation for AA and NHPI subgroups, language access, promoting inclusion, capacity building and community engagement, through enhancing research focusing on the health and well-being of AA and NHPI populations [4]. The results from a careful analysis of the RFI [7] responses and the discussions at the NIH annual AA and NHPI health research conference, May 4–5th, 2022 [8] will provide a foundation to develop actionable recommendations to the NIH leadership. It is our hope that such recommendations will have a positive impact on the AA and NHPI populations as well as other underserved ethnic and minority groups.

## Data Availability

Not applicable.
